# Radial head arthroplasty diameter impact on elbow kinematics evaluated by dynamic radiostereometric analysis

**DOI:** 10.1002/jeo2.12092

**Published:** 2024-08-08

**Authors:** Johanne F. Teilmann, Emil T. Petersen, Theis M. Thillemann, Chalotte K. Hemmingsen, Josephine O. Kipp, Maiken Stilling

**Affiliations:** ^1^ AutoRSA Research Group, Orthopedic Research Unit Aarhus University Hospital Aarhus Denmark; ^2^ Department of Clinical Medicine Aarhus University Aarhus Denmark; ^3^ Department of Orthopedic Surgery Aarhus University Hospital Aarhus Denmark

**Keywords:** elbow, kinematics, radial head arthroplasty, radial head diameter, radiostereometry

## Abstract

**Purpose:**

Radial head arthroplasty (RHA) reestablishes elbow stability after complex radial head fracture, but complication rates are high, possibly due to inappropriate implant sizing. Knowledge of impact of radial head implant diameter on elbow kinematics is limited and warranted. This study evaluated elbow kinematics of different radial head implant diameters after RHA using dynamic radiostereometric analysis (dRSA).

**Methods:**

Eight human donor arms were examined with dRSA during elbow flexion with the forearm in unloaded neutral position, and in supinated‐ and pronated position without and with 10N either varus or valgus load, respectively. Elbow kinematics were examined before and after RHA with head diameters of anatomical size, −2 mm (undersized), and +2 mm (oversized). The ligaments were kept intact by use of step‐cut humerus osteotomy for repeated RHA exchange. Bone models were obtained from CT, and by AutoRSA software bone models were matched with dRSA recordings. The elbow kinematics were described using anatomical coordinate systems.

**Results:**

Compared to the native radial head during elbow flexion, the anatomical sized RHA shifted 2.0 mm in ulnar direction during unloaded pronated forearm position. The undersized RHA shifted 1.5 mm in posterior direction and 2.1 mm in ulnar direction during unloaded pronated forearm position and increased the varus angle by 2.4° during supinated loaded forearm position. The oversized RHA shifted 1.6 mm in radial direction during loaded supinated forearm position.

**Conclusions:**

The anatomically sized RHA should be preferred as it maintained native elbow kinematics the best. The kinematic changes with oversized and undersized RHA diameters were small, suggesting forgiveness for the RHA diameter size.

**Level of Evidence:**

Level III.

**Abbreviations**
ACS: anatomical coordinate system
CT: computed tomography
dRSA: dynamic radiostereometric analysis
IOM: interosseus membrane
RHA: radial head arthroplasty
RHJ: radiohumeral joint
RSA: radiostereomtric analysis
SPM: statistical parametric mapping

## INTRODUCTION

Radial head arthroplasties (RHA) are used to treat radial head fractures where osteosynthesis is not possible and instability is present, such as terrible triad injuries and Essex‐Lopresti fractures [4, 37]. The radial head provides stability against valgus stress when the medial collateral ligament is injured, withstands axial loading with the interosseus membrane (IOM), and increases the tension in the lateral collateral ligament, thus constraining varus stress [2, 4, 17].

The reported revision rates following RHA range from 0% to 38.9% with aseptic loosening, pain, and elbow stiffness among the most frequent causes [7, 12, 18, 23, 38]. Furthermore, up to 72% of patients develop secondary osteoarthritis in the radiohumeral joint (RHJ) following primary RHA [3]. These complications may be related to the size of the RHA, and especially overstuffing has been discussed as a possible explanation [3, 24, 37].

The current concept for sizing radial head implants is to replicate the diameter and length of the native radial head [6, 28, 36]. Several studies have examined the importance of correct restoration of radial length with implantation of RHA [13, 19, 22, 27, 35, 39]. Few have studied the RHA diameter, and mainly studied factors others than the joint kinematics, such as joint pressure and IOM tension [5, 21, 33]. Sizing the radial head implant can be challenging especially in the case of a comminuted radial head fracture, which may lead to an overestimation of the diameter [9].

Oversizing the radial head diameter in RHA can increase IOM tension [21], whereas undersizing the radial head may cause joint instability but decrease the radiocapitellar joint pressure [5, 33]. Knowledge of the implications of RHA diameter on elbow joint kinematics is sparse and optimal RHA sizing remains undetermined.

Dynamic radiostereometric analysis (dRSA) is a precise imaging method to assess joint kinematics during movement and has previously been used in studying the kinematics of the wrist, elbow, shoulder, knee, and hip joints [8, 15, 16, 34].

The aim of this study was to examine the RHJ kinematics of anatomically sized, 2 mm oversized and 2 mm undersized RHA diameters compared to the native elbow, using dRSA in an experimental cadaver setting. We hypothesised that (1) the RHJ kinematics are changed from the native radial head with RHA of all diameters, and (2) the kinematic changes of the RHJ with oversized and undersized RHA compared to the native radial head are greater than the kinematic changes of the RHJ with RHA of anatomical size compared to the native radial head.

## MATERIALS AND METHODS

We have formerly investigated the kinematics in the native elbow joint and with a standard size RHA using the same material and set‐up, which is described in Hemmingsen et al. [16] The data for this study were collected jointly with the data described in Hemmingsen et al.

### Donor specimens and preparation

Eight fresh frozen human specimens including the hand to the humerus' midshaft were included in this study (mean age 82 years [range 61–89 years], five females, four right arms). The specimens were excluded if there were signs of pathologic bone abnormalities utilising fluoroscopic evaluation.

The specimens were thawed at 5°C for 48 h and prepared by removal of soft tissue and muscles, keeping the ligaments and the IOM intact. An incision was made vertically in the joint capsule on the anterior side, parallel with the lateral margin of the trochlea. A sagittal step‐cut humeral osteotomy was made with an oscillating saw for easy access to the radiocapitellar joint to exchange the radial head implants of different sizes during the study, without affecting the ligaments. Perpendicular to the osteotomy two 4.2 mm holes were drilled to allow fixation of the osteotomy with 4 mm bolts, nuts, and washers.

### Implants and surgical procedure

The Anatomic Radial Head System (Acumed) with standard stem implants was used. The system consists of five head diameters (20–28 mm), and five stem diameters (6–10 mm) with five different collar lengths (0–8 mm).

Insertion of the implants was performed according to the manufacturer's recommendations. The radial head was resected as close to the surgical neck as possible, and the bone canal was prepared using a stem reamer. To determine the anatomical implant size, the radial head diameter and the resected bone length were measured using a caliper. Additionally, a trial gauge was used to measure and verify the collar length on the stem. In the case of radial head specimens in‐between sizes, the smaller implant was always chosen. The implants were inserted with press‐fit technique in the radial bone and oriented using the laser markings on the implant.

To investigate the undersized and oversized RHA head diameter, one size (2 mm) smaller and larger head diameter than the anatomical implant size was inserted and tested. Between tests of the head diameters, the collar and stem were left in place in the radial bone canal.

### Test setup

**Figure 1:**
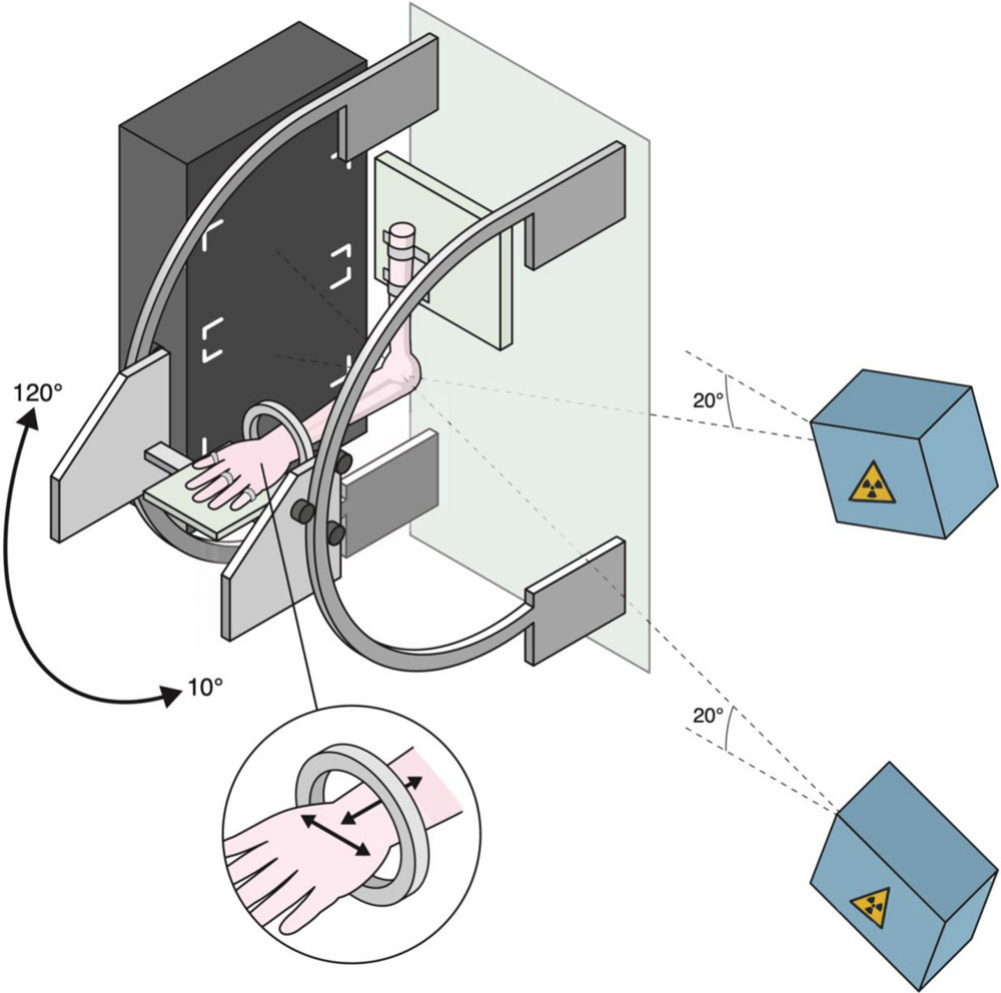
Illustration of the test setup. The donor specimen was mounted and moved in a motorised flexion‐extension motion. The X‐ray tubes (blue) were mounted to the ceiling, X‐ray detectors and calibration box (black) were placed in relation to the donor specimen for the dynamic radiostereometric recordings of the elbow motion.

A custom‐made motorised fixture was used for the movement of the specimen. The humerus was fixed to a plate on the fixture with screws. The hand was fastened with zip‐ties to another plate, which allowed low friction movement in the frontal and sagittal plane (Figure 1).

A flexion (20°–120°) elbow movement was performed with the forearm in three unloaded positions; neutral, supinated, and pronated, and two loaded positions; supinated and pronated. A load of 10N was attached to the distal ulna (3 cm proximal to the wrist joint) and applied a valgus load in the pronated forearm position and a varus load in the supinated forearm position. No axial load was applied apart from the specimens' own weight and gravity.

Dynamic RSA recordings of the elbow flexion movements in the five positions were obtained with the native radial head and with the anatomical, the undersized, and the oversized RHA.

### dRSA

Bone models of the humerus and radius were created from the segmentation of computed tomography (CT) scans (Philips Brilliance 64 CT scanner) of each specimen.

Each specimen was recorded with dRSA (AdoraRSA system, Adora RSAd, NRT X‐Ray) using two ceiling‐mounted X‐ray tubes placed vertically at 20° respective of one another and using a framerate of five frames/sec. The full flexion elbow motion included approximately 40 frames. Two digital X‐ray image detectors (Canon CXDI‐50RF) were slotted behind a uniplanar carbon fibre calibration box (Carbon box 14; Medis Specials).

A calibration image was created by averaging all the frames and calibrated using Model‐based RSA software (RSAcore, Leiden, The Netherlands). The dRSA recordings were analysed using AutoRSA software (Orthopedic Research Unit, Aarhus, Denmark). Prior to the automated registration procedure of each frame the models pose (position and orientation) was initialised. This was done manually on the first frame, while subsequent frames were initialised by extrapolating the model poses based on their optimised poses from previous frames.

The AutoRSA registration procedure included individual model registration at two levels: First, the robust optimisation scheme in half resolution, and second by a refined local optimisation in full resolution. The bone models were matched using the CT‐based bone‐volume [32]. All pixel values with an intensity above 60000 were masked out for both levels as high‐intensity values may disturb the optimisation. Additionally, for the refined optimisation the surroundings of each model were also masked out to avoid disturbance.

### Anatomical coordinate system (ACS)

**Figure 2:**
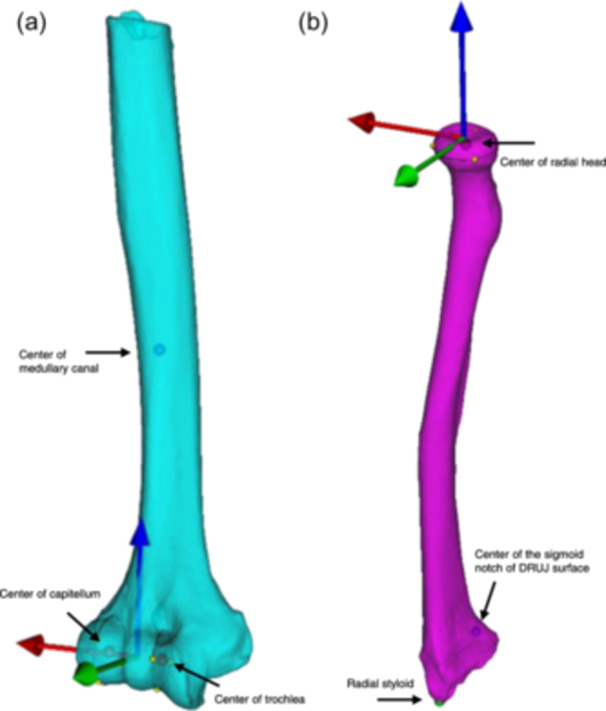
Anatomical coordinate system and landmarks: (a) Humerus (cyan): The humeral landmarks (black arrows) were the centre of trochlea and the centre of capitellum, determined by circle fits, and the centre of the medullary canal 100 mm proximal from the most distal part of the humerus. The blue arrow is the proximal‐distal axis, the red arrow is the medial‐lateral axis, and the green arrow is the anterior‐posterior axis. (b) Radius (magenta): The radial landmarks (black arrows) were the centre of the radial head, determined by circle fit, the centre of the sigmoid notch of the distal radioulnar joint surface on the radius, and the radial styloid. The blue arrow is the proximal‐distal axis, the red arrow is the medial‐lateral axis, and the green arrow is the anterior‐posterior axis.

Anatomical landmarks on the radius and humerus as described by McDonald et al. [25]. were used to create a local ACS, defined as cartesian coordinate system by three orthogonal axes (Figure 2).

For the humerus, the centre of trochlea and capitellum defined the lateral‐medial axis. An orthogonal projection from the lateral‐medial axis to the centre of the medullary canal defined the proximal‐distal axis. The cross‐product of the lateral‐medial and proximal‐distal axis defined the anterior‐posterior axis.

For the radius, the centre of the radial head and the centre of the sigmoid notch of the distal radioulnar joint surface on the radius defined the proximal‐distal direction. An orthogonal projection from the proximal‐distal axis to the radial styloid defined the anterior‐posterior axis. The cross‐product of the proximal‐distal and anterior‐posterior axis defined the lateral‐medial axis.

### Elbow joint kinematics

Using the bone‐specific ACS of the humerus and radius, the RHJ kinematics were described in six degrees of freedom using the Grood and Suntay joint coordinate system with modified equations accounting for hyper‐extension and ‐flexion [11, 14].

**Figure 3:**
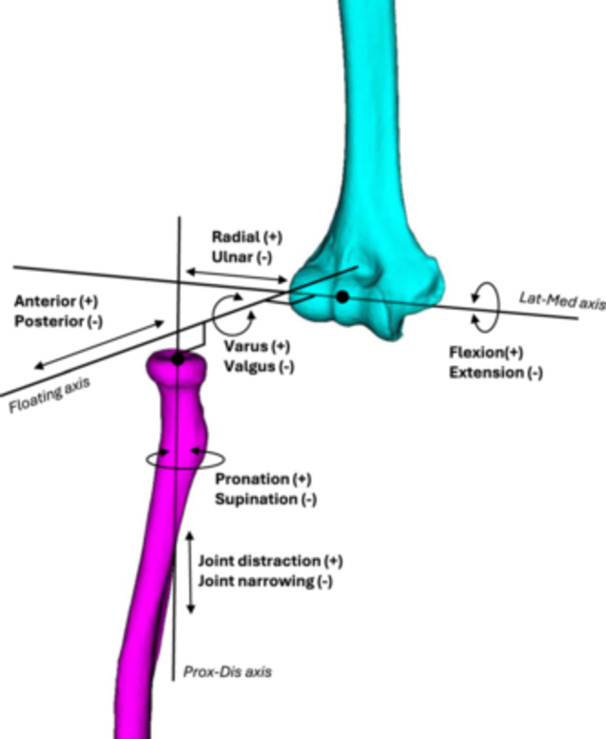
Radiohumeral joint axes and kinematic parameters referring to Grood and Suntay with modifications [11, 14]. Lat‐Med axis: Lateral‐Medial axis. Prox‐Dis axis: Proximal‐Distal axis. The black dots represent the centre of the local ACS of the radius and humerus respectively. The flexion/extension is the rotation about the lateral‐medial axis. The varus/valgus is the rotation about the floating axis (an axis mutually perpendicular to the humeral lateral‐medial axis and the radial proximal‐distal axis). The pronation/supination is the rotation about the proximal‐distal axis of the radius. The lateral/medial radial shift is the position of the radial origin on the lateral‐medial axis of the humerus. The anterior/posterior shift is the position of the radial origin on the floating axis relative to the humerus. The joint distraction/narrowing is the humeral origin's position on the radius's proximal‐distal axis.

For the RHJ the six degrees of freedom included three rotations measured in degrees: flexion(+)/extension(−), varus(+)/valgus(−), pronation(+)/supination(−), and three translations measured in millimetres: radial shift(+)/ulnar shift(−), anterior shift(+)/posterior shift(−), and joint distraction(+)/joint narrowing(−) (Figure 3). Since the rotation of the forearm was controlled in the set‐up and the RHA length was unchanged during the experiment the kinematic data for pronation/supination and joint distraction/joint narrowing were not evaluated for RHJ during the elbow flexion motion.

#### Sample size

Hemmingsen et al. [16] found the largest change in translation of the radial head in medial‐lateral direction of 0.47 (±0.26) mm. With a power of 80%, a 5% level of significance, and SD of 0.26 mm, it is possible to detect a difference of 0.31 mm in translation of the radial head with only three cadavers. Based on joint size, contact area and dynamic stability in a stable radiocapitellar joint, we expect the maximum acceptable change in translation to be 1−2 mm after insertion of a radial head prosthesis. In order to achieve a representative study population a sample size of eight cadavers was opted for.

#### Statistical analysis

Statistical parametric mapping (SPM) enables the comprehensive analysis of one‐dimensional time series for kinematic trajectories, effectively eliminating selection bias and enabling non‐directed hypothesis testing. This approach avoids the need to reduce the dataset to specific observations and mitigates the risk of false hypothesis testing caused by multiple repeated measurements [29, 31]. We utilised one‐dimensional SPM with the open‐source code spm1d (spm1d.org, v.0.4.18) [30] for Python (Python Software Foundation, v.3.10) to calculate descriptive statistics of the kinematic changes between the native elbow joint and elbow joint with RHA across the elbow flexion. A two‐sided paired Student's *t*‐test with an alpha level of 0.05 was used to compare the groups.

SPM utilises Gaussian random field theory to compute the threshold, ensuring that only the significance level of equivalently smooth Gaussian random field is exceeded under the assumption of the null hypothesis. It is assumed that the data complies with the method assumptions of SPM ‐ random, homologous, and normally distributed data. The normality was tested using QQ‐plot and D'Agostino‐Pearson K2 test [10]. For those normality tests of which reach significance at alpha level of 0.05, for example, the data did not follow normal distribution, the results of the parametric paired *t*‐test were compared to the results of the nonparametric paired *t*‐test. If differences were identified between results the nonparametric paired *t*‐test was used. The nonparametric test treats the smoothness implicit and estimates the test statistics through permutation. The parametric paired *t*‐test was used in all cases, as none of the nonparametric paired *t*‐test results deviated.

From the SPM analysis, the following kinematic differences between native radial head and radius with RHA for all sizes and forearm positions were extracted: the starting difference (at 20° of elbow flexion), the end difference (at 120° of elbow flexion), the minimal difference at which degree of elbow flexion, and the maximum difference at which degree of elbow flexion. The random variation of the means was described as 95% confidence intervals (CIs) in graphs and tables.

## RESULTS

Standard deviations can be viewed in the Supporting Information S1: Tables S1–S3.

The following kinematic results are described as maximum motion differences (mean [CI 95%]) for the RHJ motion with the native radial head compared to the RHJ motion with RHA in different head diameters.

**Figure 4:**
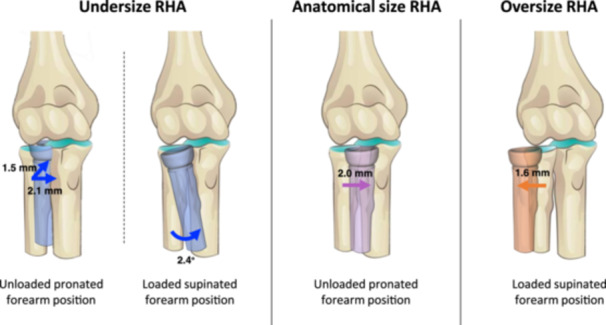
Illustration of the summarised maximum kinematic differences during elbow flexion motion between the elbow with native radial head compared to motion of the elbow with radial head arthroplasty (RHA) of three different sizes. The observed maximum was not necessarily found in full extension as shown in this figure. From left to right: The undersized RHA shifted the radius 1.5 mm in posterior direction and 2.1 mm in ulnar direction in the unloaded pronated forearm position and increased varus angle of 2.4° in the loaded supinated forearm position. The anatomical RHA shifted the radius 2.0 mm in ulnar direction in the unloaded pronated position. The oversized RHA shifted the radius 1.6 mm in radial direction in the loaded supinated forearm position. Colour codes correspond to Figures 3, 4, 5, Tables 2 and 3. The migrations showed by the coloured models are not scaled or corrected with the listed translations in mm or rotations in degrees.

The most important findings are summarised in the illustration (Figure 4) and the text below. The unloaded supinated forearm position and loaded pronated forearm position revealed kinematic changes between the native radial head and all RHA sizes comparable to their loaded and unloaded equivalents (graphs are available in Supporting Information S1: Figures S1 and S2).

### Neutral forearm position

**Figure 5:**
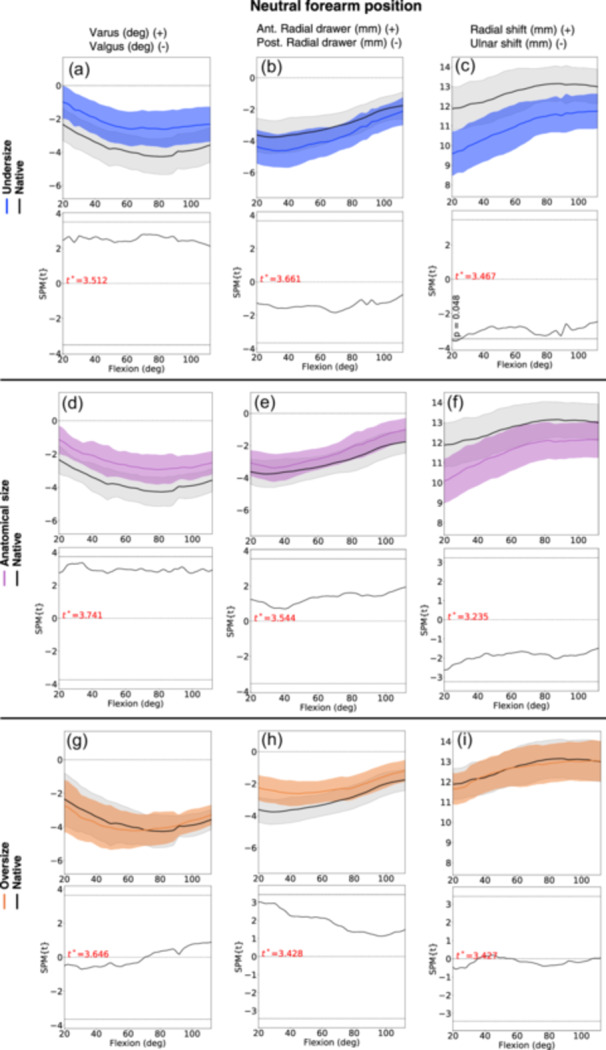
Kinematic results (statistical parametric mapping [SPM] graphs) for radial head arthroplasty (RHA) of undersize (graph a−c, blue line), anatomical size (graph d−f, purple line), and oversize (graph g−i, orange line) compared to the native radial head (black line), with the forearm in neutral position. The faded areas around lines represent confidence interval (CI) 95%. The graph below each SPM graph presents the post hoc scalar field *t*‐tests (SnPM{t}), depicting any statistical difference in the kinematics (grey areas) between the native radial head and the RHA during the elbow flexion motion. The critical threshold of significance is indicated by the thin black line. In these plots, there are no statistically significant differences between the line graphs.

No statistically significant kinematic change was observed in all directions (varus/valgus, anterior/posterior drawer, and radial/ulnar shift) between the native radial head compared with all RHA sizes (Figure 5, Table 1).

**Table 1:** Kinematic differences between native radial head and radius with RHA in neutral forearm position.

	20° flexion angle (95% CI)	120° flexion angle (95% CI)	Min. (95% CI) at which flexion angle	Max. (95% CI) at which flexion angle
Varus(+)/valgus(−) (deg)				
Undersize	1.4 (−0.6; 3.3)	1.2 (−0.8; 3.3)	1.3 (−0.8; 3.3) at 112°	1.7 (−0.4; 3.8) at 78°
Anatomical	1.2 (−0.4; 2.8)	1.1 (−0.3; 2.4)	1.1 (−0.3; 2.4) at 112°	1.4 (−0.5; 3.3) at 49°
Oversize	−0.4 (−3.5; 2.6)	0.3 (−0.9; 1.5)	0.0 (−1.8; 1.8) at 71°	−0.5 (−3.2; 2.1) at 31°
Anterior(+)/posterior(−) (mm)				
Undersize	−0.7 (−2.9; 1.4)	−0.4 (−2.1; 1.4)	−0.4 (−2.1; 1.4) at 112°	−0.9 (−3.1; 1.3) at 40°
Anatomical	0.5 (−1.0; 2.1)	0.8 (−0.6; 2.2)	0.3 (−1.3; 2.0) at 40°	0.8 (−0.6; 2.2) at 112°
Oversize	1.4 (−0.2; 2.9)	0.6 (−0.7; 1.9)	0.5 (−1.0; 1.9) at 97°	1.4 (−0.2; 2.9) at 20°
Radial(+)/ulnar(−) (mm)				
Undersize	−2.3 (−4.5; −0.0)	−1.2 (−3.0; 0.5)	−1.2 (−3.0; 0.5) at 112°	−2.3 (−4.5; −0.1) at 21°
Anatomical	−1.8 (−4.0; 0.4)	−0.8 (−2.6; 1.0)	−0.8 (−2.6; 1.0) at 112°	−1.8 (−4.0; 0.4) at 20°
Oversize	−0.2 (−1.7; 1.3)	0.0 (−1.9; 2.0)	0.0 (−1.8; 1.8) at 57°	−0.3 (−1.8; 1.3) at 22°

*Note*: Mean and 95% confidence intervals (CIs) of the differences between the native radial head and the radial head arthroplasty (RHA) with undersized, anatomical and oversized diameter for the kinematic parameters: varus/valgus angle, anterior/posterior shift and radial/ulnar shift. The 20° flexion angle represents kinematic difference at the start of the elbow flexion motion, and the 120° flexion angle represents the end of the elbow flexion motion. The smallest (min.) and largest (max.) difference between the native radial head and the radius with RHA are also presented.

The undersized and the anatomical sized RHA tended to shift in ulnar direction and towards varus angulation during the elbow flexion motion. For the undersized RHA, the ulnar shift was 2.3 mm (CI 95% −4.5; −0.1) and the mean varus angle 1.7° (CI 95% −0.4; 3.8). For the anatomical size RHA, the mean ulnar shift was 1.8 mm (CI 95% −4.0; 0.4), and the mean varus angle 1.4° (CI 95% −0.5; 3.3).

### Pronated forearm position

**Figure 6:**
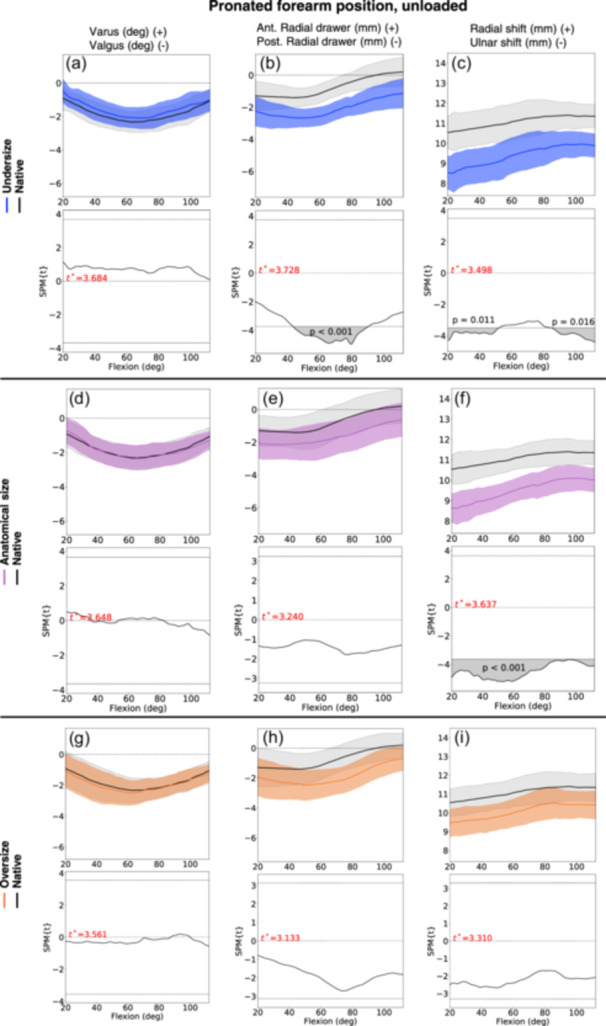
Kinematic results (statistical parametric mapping [SPM] graphs) for radial head arthroplasty (RHA) of undersize (graph a−c, blue line), anatomical size (graph d−f, purple line), and oversize (graph g−i, orange line) compared to the native radial head (black line), with the forearm in unloaded pronated forearm position. The faded areas around lines represent confidence interval (CI) 95%. The graph below each SPM graph presents the post hoc scalar field *t*‐tests (SnPM{t}), depicting any statistical difference in the kinematics (grey areas) between the native radial head and the RHA during the elbow flexion motion. The critical threshold of significance is indicated by the thin black line. The faded grey areas represent statistically significant differences between the line graphs.

In the unloaded pronated forearm position, the anatomical‐sized RHA shifted the radius 2.0 mm (CI 95% −3.5; −0.4) in ulnar direction compared to the native radial head. The undersized RHA shifted 2.1 mm (CI 95% −3.9; −0.2) in ulnar direction and 1.5 mm (CI 95% −2.7; −0.4) in posterior direction (Figure 6, Table 2).

**Table 2:** Kinematic differences between native radial head and radius with RHA in pronated forearm position.

	20° flexion angle (95% CI)	120° flexion angle (95% CI)	Min. (95% CI) at which flexion angle	Max. (95% CI) at which flexion angle
Unloaded				
Varus(+)/valgus(−) (deg)				
Undersize	0.4 (−1.0; 1.8)	0.0 (−1.3; 1.3)	0.0 (−1.3; 1.3) at 112°	0.4 (−1.0: 1.8) at 20°
Anatomical	0.2 (−1.3; 1.7)	−0.4 (−1.3; 0.8)	0.0 (−1.4; 1.4) at 82°	−0.2 (−1.3; 0.8) at 112°
Oversize	−0.2 (−2.4; 2.0)	−0.2 (−1.4; 1.0)	0.0 (−1.4; 1.4) at 100°	−0.2 (−2.3; 1.9) at 43°
Anterior(+)/posterior(−) (mm)				
Undersize	−1.0 (−2.8; 0.8)	−1.3 (−3.2; 0.5)	−1.0 (−2.8; 0.8) at 20°	−1.5 (−2.7; −0.4) at 80°
Anatomical	−0.8 (−2.6; 1.1)	−0.8 (−2.9; 1.2)	−0.7 (−2.8; 1.4) at 49°	−1.1 (−3.2; 1.0) at 83°
Oversize	−0.6 (−3.2; 1.9)	−0.9 (−2.5; 0.7)	−0.6 (−3.2; 1.9) at 20°	−1.5 (−3.3; 0.2) at 74°
Radial(+)/ulnar(−) (mm)				
Undersize	−2.0 (−3.7; −0.4)	−1.5 (−2.6; −0.3)	−1.4 (−2.6; −0.2) at 103°	−2.1 (−3.9; −0.2) at 23°
Anatomical	−1.9 (−2.2; −0.5)	−1.3 (−2.5; −0.2)	−1.2 (−2.5; −0.0) at 101°	−2.0 (−3.5; −0.4) at 23°
Oversize	−1.1 (−2.6; 0.3)	−0.9 (−2.4; 0.5)	−0.8 (−2.5; 0.8) at 86°	−1.2 (−2.6; 0.3) at 43°
Valgus loaded				
Varus(+)/valgus(−) (deg)				
Undersize	−0.0 (−1.5; 1.5)	−0.9 (−2.5; 0.7)	−0.0 (−1.5; 1.5) at 20°	−1.0 (−2.4; 0.5) at 108°
Anatomical	0.2 (−2.3; 2.6)	−0.3 (−1.3; 0.6)	−0.0 (−2.0; 2.0) at 35°	−0.4 (−1.7; 0.9) at 80°
Oversize	−0.2 (−2.2; 1.9)	−0.5 (−2.1; 1.1)	−0.0 (−1.9; 1.8) at 21°	−0.9 (−2.7; 0.9) at 55°
Anterior(+)/posterior(−) (mm)				
Undersize	−0.7 (−2.5; 1.1)	−1.0 (−2.7; 0.7)	−0.6 (−2.2; 1.0) at 39°	−1.0 (−2.7; 0.7) at 104°
Anatomical	−0.9 (−2.8; 1.1)	−0.6 (−2.5; 1.3)	−0.6 (−2.5; 1.3) at 112°	−0.9 (−3.0; 1.1) at 66°
Oversize	−1.0 (−3.8; 1.8)	−0.5 (−1.9; 0.8)	−0.5 (−1.9; 0.8) at 111°	−1.1 (−3.4; 1.2) at 48°
Radial(+)/ulnar(−) (mm)				
Undersize	−1.1 (−2.5; 0.2)	−1.1 (−2.6; 0.4)	−0.8 (−2.5; 0.8) at 56°	−1.1 (−2.5; 0.2) at 20°
Anatomical	−1.4 (−2.6; −0.2)	−1.2 (−2.5; 0.1)	−0.9 (−2.2; 0.3) at 54°	−1.4 (−2.8; −0.0) at 28°
Oversize	−0.7 (−1.9; 0.4)	−0.7 (−2.2; 0.7)	−0.4 (−1.6; 0.8) at 57°	−0.9 (−2.4; 0.6) at 27°

*Note*: Mean and 95% confidence intervals (CIs) of the differences between the native radial head and the RHA with undersized, anatomical and oversized diameter for the kinematic parameters: varus/valgus angle, anterior/posterior shift and radial/ulnar shift. The 20° flexion angle represents kinematic difference at the start of the elbow flexion motion, and the 120° flexion angle represents the end of the elbow flexion motion. The smallest (min.) and largest (max.) difference between the native radial head and the RHA are also presented. The colour code corresponds to the colour of the radius with RHA in Figures 3, 4, 5, 6.

In the loaded pronated forearm position, no statistically significant kinematic changes were observed to the varus‐valgus angle between all three RHA sizes compared to the native radial head. There was a tendency for all three RHA sizes to present a posterior shift (range 0.9–1.1 mm) and an ulnar shift (range 0.9–1.4 mm).

### Supinated forearm position

**Figure 7:**
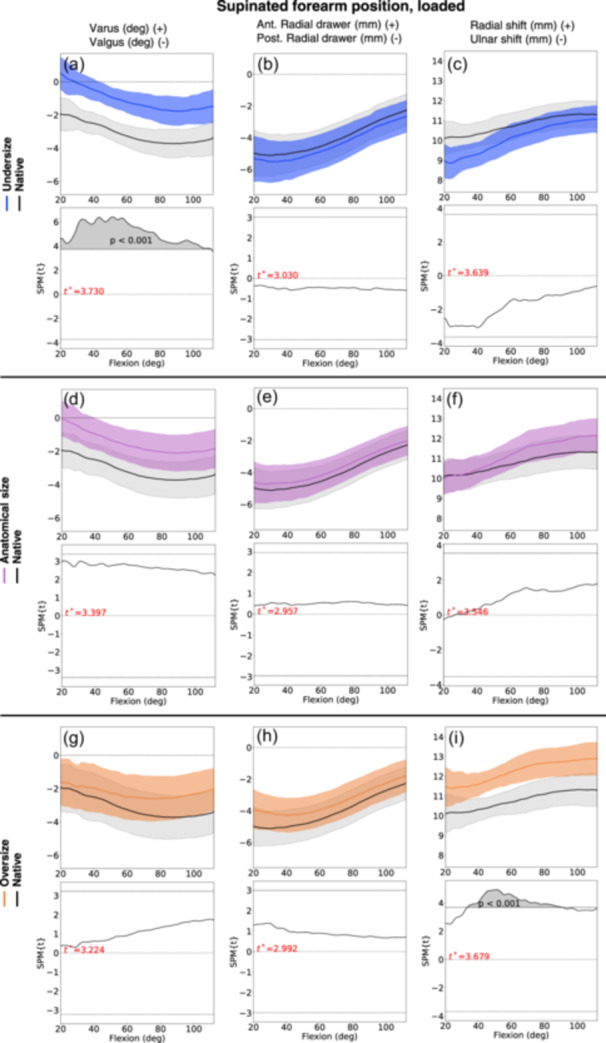
Kinematic results (statistical parametric mapping [SPM] graphs) for radial head arthroplasty (RHA) of undersize (graph a−c, blue line), anatomical size (graph d−f, purple line), and oversize (graph g−i, orange line) compared to the native radial head (black line), with the forearm in loaded supinated forearm position. The faded areas around lines represent confidence interval (CI) 95%. The graph below each SPM graph presents the post hoc scalar field *t*‐tests (SnPM{t}), depicting any statistical difference in the kinematics (grey areas) between the native radial head and the RHA during the elbow flexion motion. The critical threshold of significance is indicated by the thin black line. The faded grey areas represent statistically significant differences between the line graphs.

The oversized RHA shifted the radius 1.6 mm (CI 95% 0.2; 3.0) in radial direction during the loaded supinated forearm position (Figure 7, Table 3), and mean 2.0 mm (CI 95% 0.1; 3.8) in the unloaded supinated forearm position. With decreasing RHA diameter, the radius tended to shift in ulnar direction, though not statistically significantly different from the kinematics of the native radial head.

**Table 3:** Kinematic differences between native radial head and radius with RHA in supinated forearm position.

	20° flexion angle (95% CI)	120° flexion angle (95% CI)	Min. (95% CI) at which flexion angle	Max. (95% CI) at which flexion angle
Unloaded				
Varus(+)/valgus(−) (deg)				
Undersize	1.2 (−0.5; 2.9)	1.5 (−0.4; 3.4)	0.9 (−0.7; 2.6) at 60°	1.7 (−0.2; 3.5) at 95°
Anatomical	1.1 (−0.4; 2.5)	1.0 (−0.9; 2.8)	0.8 (−1.1; 2.7) at 61°	1.1 (−0.4; 2.6) at 41°
Oversize	−0.0 (−2.4; 2.4)	0.6 (−1.1; 2.3)	0.0 (−2.4; 2.4) at 21°	0.7 (−1.1; 2.5) at 108°
Anterior(+)/posterior(−) (mm)				
Undersize	−0.6 (−3.6; 2.4)	−0.6 (−3.2; 2.1)	−0.6 (−3.1; 2.0) at 96°	−1.2 (−4.8; 2.3) at 53°
Anatomical	0.3 (−2.6; 3.3)	0.3 (−1.9; 2.6)	−0.0 (−3.1; 3.0) at 54°	0.5 (−1.5; 2.6) at 85°
Oversize	1.3 (−1.6; 4.2)	0.4 (−2.3; 3.1)	0.4 (−2.3; 3.1) at 112°	1.3 (−1.6; 4.2) at 20°
Radial(+)/ulnar(−) (mm)				
Undersize	−0.6 (−2.5; 1.3)	0.3 (−1.0; 1.5)	−0.0 (−1.4; 1.4) at 70°	−0.7 (−1.9; 1.4) at 37°
Anatomical	0.4 (−1.4; 2.2)	1.2 (−0.4; 2.8)	0.3 (−1.5; 2.1) at 25°	1.2 (−0.4; 2.8) at 111°
Oversize	1.3 (−0.5; 3.0)	1.9 (0.1; 3.6)	1.1 (−0.3; 2.6) at 33°	2.0 (0.1; 3.8) at 108°
Varus loaded				
Varus(+)/valgus(−) (deg)				
Undersize	2.4 (0.5; 4.4)	1.9 (−0.1; 3.9)	1.9 (−0.1; 3.9) at 112°	2.4 (0.5; 4.4) at 20°
Anatomical	1.8 (−0.3; 4.0)	1.5 (−0.8; 3.9)	1.5 (−0.4; 3.3) at 48°	1.9 (−0.2; 4.0) at 21°
Oversize	0.3 (−2.5; 3.1)	1.3 (−1.2; 3.9)	0.2 (−2.4; 2.9) at 29°	1.4 (−1.1; 3.9) at 110°
Anterior(+)/posterior(−) (mm)				
Undersize	−0.3 (−3.2; 2.5)	−0.4 (−2.4; 1.6)	−0.3 (−3.1; 2.4) at 24°	−0.5 (−3.1; 2.4) at 24°
Anatomical	0.4 (−2.2)	0.3 (−1.6; 2.1)	0.3 (−1.6; 2.1) at 112°	0.4 (−1.9; 2.7) at 31°
Oversize	1.1 (−1.4; 3.6)	0.5 (−1.6; 2.5)	0.5 (−1.6; 2.5) at 99°	1.1 (−1.4; 3.6) at 20°
Radial(+)/ulnar(−) (mm)				
Undersize	−1.2 (−2.8; 0.5)	−0.2 (−1.6; 1.1)	−0.2 (−1.6; 2.5) at 112°	0.0 (−2.9; 0.3) at 27°
Anatomical	1.8 (−0.3; 4.0)	1.5 (−0.8; 3.9)	1.5 (−0.4; 3.3) at 48°	1.9 (−0.2; 4.0) at 21°
Oversize	1.4 (−0.6; 3.3)	1.6 (−0.0; 3.3)	1.2 (−0.5; 2.9) at 24°	1.6 (0.2; 3.0) at 69°

*Note*: Mean and 95% confidence intervals (CIs) of the differences between the native radial head and the radial head arthroplasty (RHA) with undersized, anatomical and oversized diameter for the kinematic parameters: varus/valgus angle, anterior/posterior shift and radial/ulnar shift. The 20° flexion angle represents kinematic difference at the start of the elbow flexion motion, and the 120° flexion angle represents the end of the elbow flexion motion. The smallest (min.) and largest (max.) difference between the native radial head and the RHA are also presented. The colour code corresponds to the colour of the radius with RHA in Figures 3, 4, 5, 6.

For the undersized RHA, a varus angle of mean 2.4° (CI 95% 0.5; 4.4) was observed during varus loading in supinated forearm position (Figure 7, Table 3). Likewise, the anatomical size RHA tended to have a varus angle during varus load (mean 1.9° [CI 95% −0.2; 4.0]).

No statistically significant kinematic change in varus‐valgus angle was observed between the native radial head and the oversized RHA. No statistically significant kinematic differences in anterior‐posterior translation were observed between the native radial head and RHA of all sizes.

## DISCUSSION

We investigated the RHJ kinematics during elbow flexion motion in three forearm positions and found only small kinematic differences between the native radial head and RHA in three diameter sizes. In the neutral forearm position, no statistically significant differences were observed. In the loaded supinated forearm position compared to the native elbow, we observed a 2.4° increased varus angle of the radius with undersized RHA and a 1.6 mm radial shift of the radius with an oversized RHA. In the unloaded pronated forearm position, we observed a 2.0 mm ulnar shift of the radius with an anatomical RHA, and a 2.1 mm ulnar shift and a 1.5 mm posterior shift of the radius with an undersized RHA.

### Undersized RHA diameter

The undersized RHA displayed the most kinematic differences of the three investigated RHA sizes compared to the native radial head during the elbow flexion motion. This may be due to the systematic practice of choosing the smaller size for the anatomically sized RHA diameter when in between RHA size options, so increased laxity with the undersized implant was expected. The observed displacement of radial‐ulnar shift for the anatomical size RHA and the undersized RHA was also expected and corresponded nicely to the decrease in the RHA head diameter.

Our findings suggest that RHA diameter does not affect valgus stability, whereas the varus stability was more sensitive to a change from the native radial head size and to decreased RHA size. However, the changes towards a varus angle with RHA diameters different from the native radial head size were small and may not be clinically relevant. Other studies suggest that ligament stability has a greater effect on elbow varus/valgus stability than RHA size. In a cadaveric study, Jensen et al. showed that lateral collateral ligament status has a bigger impact on elbow varus angle (laxity) than radial head excision or RHA [17]. In this study, Jensen et al. found that isolated radial head excision increased the varus angle by mean 4.8°, and an isolated lateral collateral ligament excision increased varus angle by mean 14.1°. In a review by Kodde et al. [20] the authors argue that instability after RHA is more often due to ligamentous injuries or fractures being either unassessed or in bad condition, rather than the size of the implant. As the elbow ligaments in our study remained intact, we studied only the kinematic effect of RHA diameter within a clinically interesting range from the native radial head size (±2 mm), and our data suggest such variation in RHA head size to have only a small effect on elbow kinematics.

Songy et al. [33] investigated posterolateral rotatory elbow instability with different RHA diameters under valgus stress in a cadaver study. They used the same three RHA diameters (20, 22, and 24 mm) for all cadaveric elbows, which meant that several RHAs were two or more RHA sizes undersized compared to the native radial head. This may explain why Songy et al. found an increased posterior translation in supinated forearm position with decreasing radial head arthroplasty diameter, which was significant only for the smallest diameter (20 mm). A comminuted fracture can impact the measurement of the native radial head diameter, but Abdulla et al. suggest that the misestimation is likely within 1 mm, which is why we studied only ±2 mm (one RHA head size) from the native radial head [1]. Songy et al. found that the larger RHA diameters more closely resembled the native radial head diameter size and kinematics in terms of posterior translation. This corresponds to our results of the anatomical size implant retaining native kinematics the best.

### Oversized RHA diameter

The observed radial translation for the oversized RHA was caused by contact with the ulna sigmoid notch and constraints in the radioulnar joint. This did not measurably affect varus/valgus angulation nor anterior‐posterior translation of the RHJ.

To our knowledge, this is the first study examining elbow kinematics with RHA of oversized diameter. The lack of literature may reflect a normal clinical practice to undersize the RHA diameter, when the native radial head is in‐between sizes. Mirzayan et al. [26] studied 405 patients operated with RHA for traumatic radial head fracture and found increasing RHA diameter to be correlated with the risk of revision compared to the smallest RHA diameter. Despite the wide 95% CI for the difference, they concluded that the largest RHA diameters might be unnecessary to fulfil the purpose of the implant. It is not known if unintentional oversizing of the RHA diameter has occurred, but the study by Mirzayan et al. indicate that oversized RHA diameter is a clinical disadvantage. Our results suggest that oversizing the RHA diameter by one size (+2 mm) larger than the native radial head induce only minor changes to the RHJ in our cadaver setup.

### Other factors

Likely, there are other factors than radial head dimeter size, which can influence the elbow joint kinematics and the overall success of a RHA implant. Lanting et al. [21] showed that tension on the IOM increases with increasing RHA diameter, and Bachman et al. [5] found that undersizing the RHA diameter by 2 mm lowered the peak pressure of the radiocapitellar joint. We have formerly studied the relation of IOM tension, radiocapitellar joint peak pressure and elbow kinematics of an anatomical size RHA in comparison with a native radial head [16]. In this study, we found that the joint peak pressure and the tension of the IOM increased with insertion of an anatomical‐sized RHA compared to the elbow with native radial head. However, these kinematic changes were in the submillimeter range and potentially not clinically relevant.

Undersizing the RHA diameter by one size (2 mm) compared to the native radial head likely leaves the IOM tension and radiocapitellar joint pressure at normal or subnormal levels and result in permissible kinematic changes. As shown in the present study, undersizing the RHA diameter by one size seems appropriate.

The length of the radial neck and head segment may be very difficult to measure in a fracture situation. A length difference from the normal may also affect elbow kinematics but was not evaluated in the present study. In a cadaver study, Van Glabbeek et al. found significantly altered elbow joint kinematics (static point‐evaluation set‐up) and increased joint pressure with RHA that overlengthened the radius by 2.5 mm. Likewise, 2.5 mm under lengthened RHA altered the elbow kinematics but did not increase the joint pressure. Thus, restoring the length of the radius within 2.5 mm is advisable. In addition, overstuffing with a large RHA is associated with development of osteoarthritis of the elbow joint [3]. No dynamic studies during a full elbow flexion motion have been performed to study the kinematic consequence of overlengthening the radius with insertion of RHA. Thus, further research is needed to investigate the kinematic influence of radial head length.

### Strengths and limitations

The main strength of this study is the use of highly precise methods in terms of dynamic RSA combined with CT bone models for analysis of elbow kinematics with the AutoRSA software [8]. Furthermore, the full kinematic trajectory of an elbow flexion motion was examined rather than static point evaluation. Dynamic RSA could also be used in the clinical setting, to evaluate the elbow joint kinematics before and after RHA surgery in patients, which could help understand the cause of motion restrictions and functional problems.

Another strength in the study setup is the preservation of the ligaments of the elbow specimens through use of a step‐cut osteotomy, which enabled analysis of the kinematic effect of only the RHA diameter.

Limitations of this study include the age of the cadaveric donor specimens, being older than the age of the patients typically treated with RHA. In addition, the limited sample size might amplify bias related to individual variability.

The annulare ligament and the joint capsule was not opened during the recordings of the elbow with the native radial head, and after insertion of RHA we did not suture the annular ligament and joint capsule, which could have influenced the results in our study. Furthermore, the muscles and tendons of the cadaver specimens were removed to improve radiocapitellar joint access and correct size measurement of the RHA. In the clinical setting, the kinematics would most likely be affected by muscle tension, but this could not be accounted for in this cadaveric study.

## CONCLUSION

We found that the kinematics of the RHJ changed a few mm and degrees when inserting a 2 mm undersized and 2 mm oversized RHA in comparison with the native radial head during elbow flexion. The anatomical‐sized RHA provided kinematics comparable to the native joint and should therefore be preferred. However, this study suggests some forgiveness with a RHA head size variation of ±2mm as compared to the native radial head in terms of elbow joint kinematics.

## AUTHOR CONTRIBUTIONS

**Johanne F. Teilmann**: Substantial contributions to analysis and interpretation of data; drafted the paper. **Emil T. Petersen**: Substantial contribution to analysis and interpretation of data; revised the paper critically. **Theis M. Thillemann**: Substantial contributions to research design, the acquisition, and interpretation of data; revised the paper critically. **Chalotte K. Hemmingsen**: Substantial contributions to research design and the acquisition of data. **Josephine O. Kipp**: Substantial contributions to interpretation of data; revised the paper critically. **Maiken Stillin**g: Substantial contributions to research design, the acquisition, and interpretation of data. Revised the paper critically. This manuscript has been read and approved by all authors and all authors confirm that the manuscript represents honest scientific work.

## CONFLICT OF INTEREST STATEMENT

The authors declare no conflict of interest.

## ETHICS STATEMENT

Relevant approvals were obtained from the Central Denmark Region Committee on Health Research Ethics (case number 1‐16‐02‐370‐16, issued 24 February 2016).

## Supporting information

Supporting information.

## Data Availability

Due to legal restrictions, research data are not shared.
