# Supplementary material for: Radial head arthroplasty diameter impact on elbow kinematics evaluated by dynamic radiostereometric analysis
Source: J Exp Orthop. 2024 Aug 8;11(3):e12092. doi: 10.1002/jeo2.12092 (PMC11306918; doi:10.1002/jeo2.12092)
Supplement: Supplementary file 1 — Supporting information. [file JEO2-11-e12092-s001.docx]

# Supplementary information

**Supporting Fig. 1**. Kinematic results (SPM graphs) for the radius with RHA of all sizes (colored lines) compared to the native radial head (black line), with the forearm in loaded pronated forearm position. The faded areas around lines represent CI 95%. The graph below each SPM graph presents the post hoc scalar field t tests (SnPM{t}), depicting any statistical difference in the kinematics (gray areas) between the native radial head and the radius with RHA during the elbow flexion-extension motion. The critical threshold of significance is indicated by the thin black line. In these plots, there are no statistically significant differences between the line graphs.

#
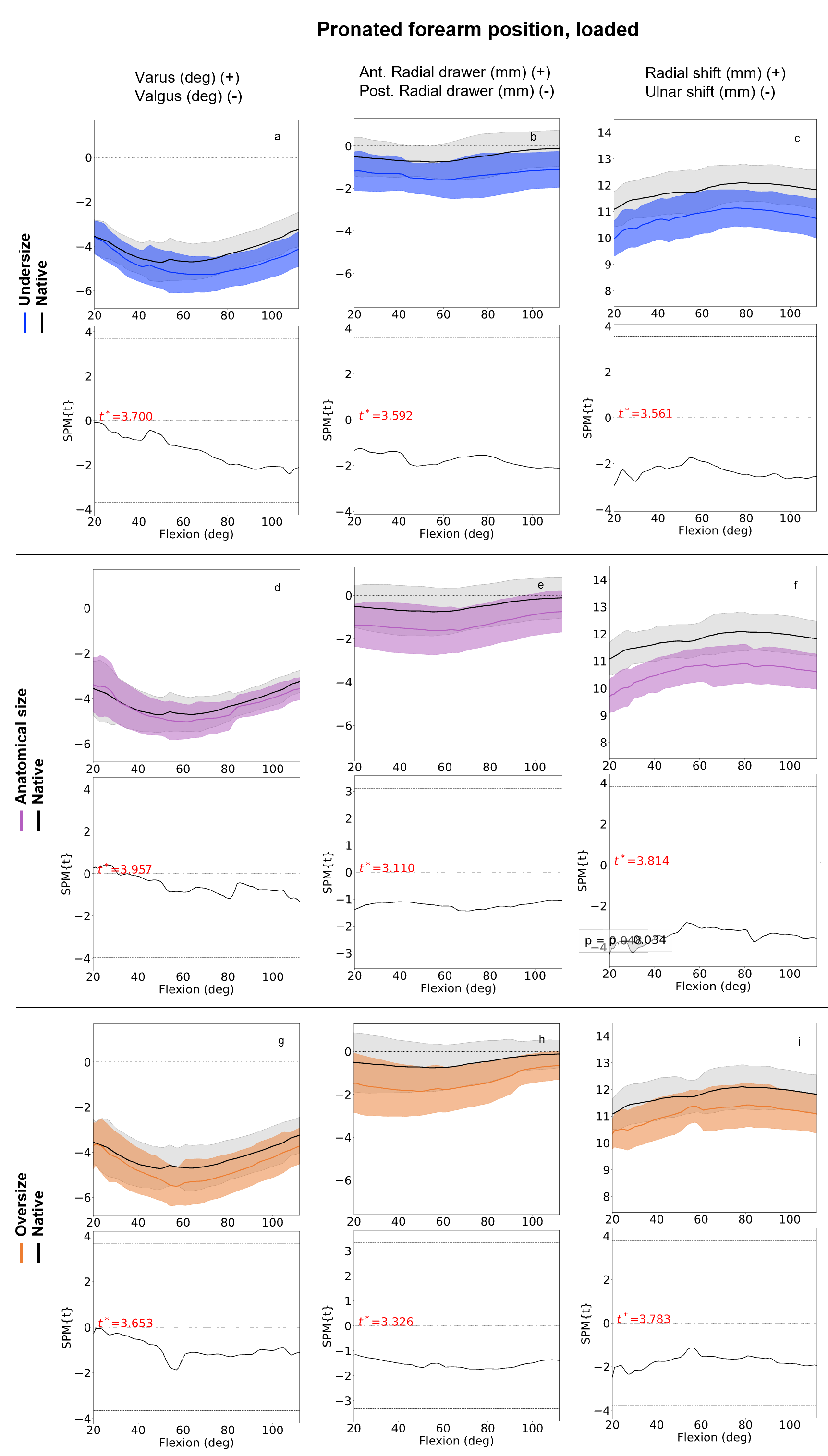


**Supporting Fig. 2**. Kinematic results (SPM graphs) for the radius with RHA of all sizes (colored lines) compared to the native radial head (black line), with the forearm in unloaded supinated forearm position. The faded areas around lines represent CI 95%. The graph below each SPM graph presents the post hoc scalar field t tests (SnPM{t}), depicting any statistical difference in the kinematics (gray areas) between the native radial head and the radius with RHA during the elbow flexion-extension motion. The critical threshold of significance is indicated by the thin black line. The faded grey areas represent statistically significant differences between the line graphs.


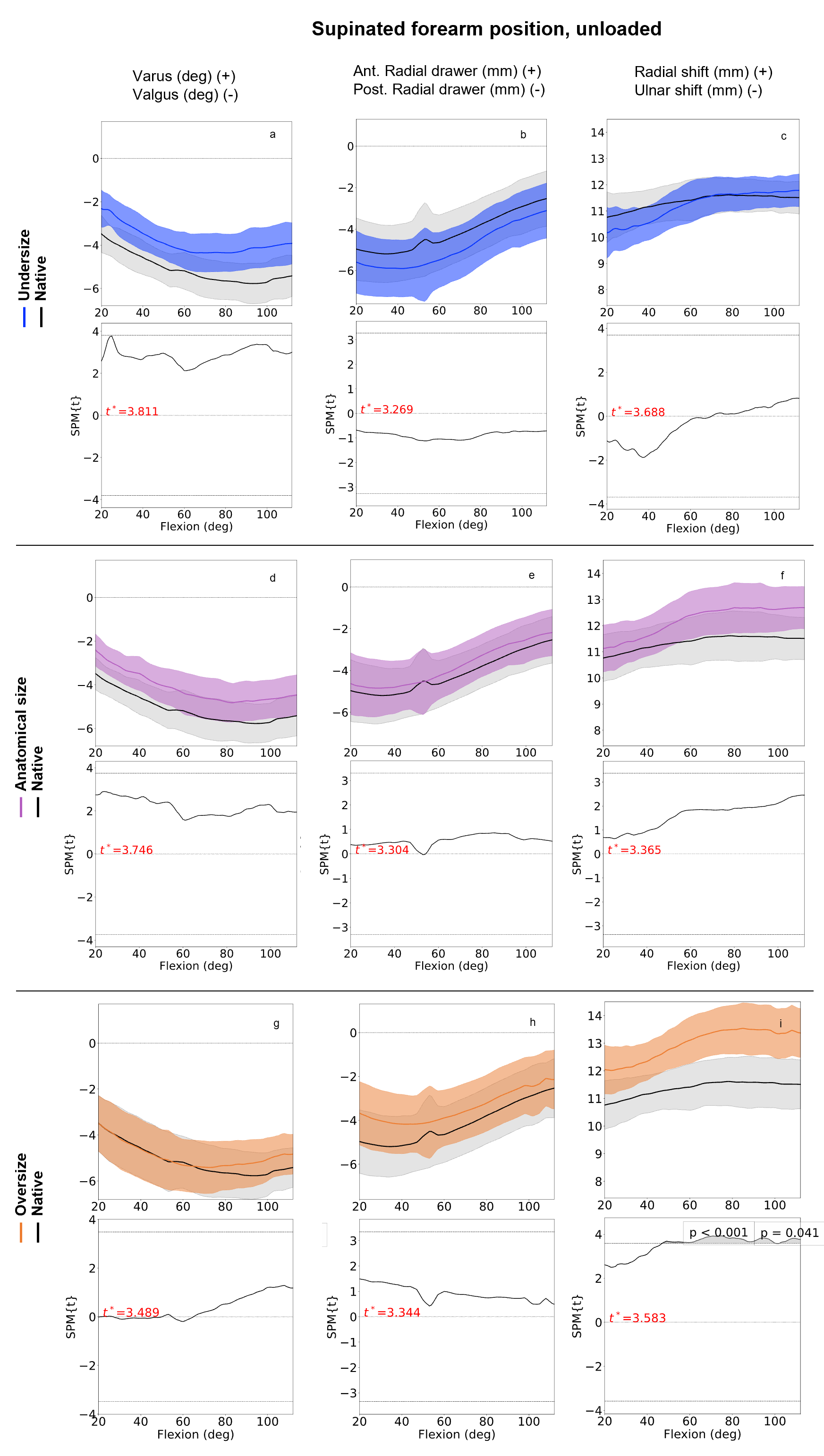


**Supporting table 1. Kinematic differences between native radial head and radius with RHA in neutral forearm position.**

Mean and standard deviation of the differences between the native radial head and the radius with RHA with undersized, anatomical and oversized diameter for the kinematic parameters: varus/valgus angle, anterior/posterior shift and radial/ulnar shift. The 20° flexion angle represents kinematic difference at the start of the elbow flexion-extension motion, and the 120° flexion angle represents the end of the elbow flexion-extension motion. The smallest (min.) and largest (max.) difference between the native radial head and the radius with RHA are also presented.

|  | **20° flexion angle (SD)** | **120° flexion angle (SD)** | **Min. (SD) at which flexion angle** | **Max. (SD) at which flexion angle** |
| --- | --- | --- | --- | --- |
| ***Varus/valgus (deg)*** |  |  |  |  |
| *Undersize* | 1.4 (3.4) | 1.2 (2.4) | 1.3 (2.8) at 112° | 1.7 (2.9) at 78° |
| *Anatomical* | 1.2 (1.5) | 1.1 (1.5) | 1.1 (1.0) at 112° | 1.4 (2.0) at 49° |
| *Oversize* | -0.4 (5.7) | 0.3 (5.7) | 0.0 (2.0) at 71° | -0.5 (4.3) at 31° |
| ***Anterior/posterior (mm)*** |  |  |  |  |
| *Undersize* | -0.7 (2.7) | -0.4 (2.7) | -0.4 (1.8) at 112° | -0.9 (2.9) at 40° |
| *Anatomical* | 0.5 (1.6) | 0.8 (1.6) | 0.3 (1.8) at 40° | 0.8 (1.3) at 112° |
| *Oversize* | 1.4 (1.4) | 0.6 (1.6) | 0.5 (1.4) at 97° | 1.4 (1.6) at 20° |
| ***Radial/ulnar (mm)*** |  |  |  |  |
| *Undersize* | -2.3 (3.3) | -1.2 (3.3) | -1.2 (2.0) at 112° | -2.3 (3.2) at 21° |
| *Anatomical* | -1.8 (3.7) | -0.8 (3.7) | -0.8 (2.5) at 112° | -1.8 (3.7) at 20° |
| *Oversize* | -0.2 (1.6) | 0.0 (1.6) | 0.0 (2.3) at 57° | -0.3 (1.6) at 22° |

**Supporting table 2. Kinematic differences between native radial head and radius with RHA in pronated position.**

Mean and standard deviation of the differences between the native radial head and the radius with RHA with undersized, anatomical and oversized diameter for the kinematic parameters: varus/valgus angle, anterior/posterior shift and radial/ulnar shift. The 20° flexion angle represents kinematic difference at the start of the elbow flexion-extension motion, and the 120° flexion angle represents the end of the elbow flexion-extension motion. The smallest (min.) and largest (max.) difference between the native radial head and the radius with RHA are also presented. The color code corresponds to the color of the radius with RHA in figure 3, 4, 5 and 6.

|  | | **20° flexion angle (SD)** | **120° flexion angle (SD)** | **Min. (SD) at which flexion angle** | **Max. (SD) at which flexion angle** |
| --- | --- | --- | --- | --- | --- |
| **Unloaded** | |  |  |  |  |
|  | ***Varus(+)/valgus(-) (deg)*** |  |  |  |  |
|  | *Undersize* | 0.4 (1.2) | 0.0 (1.2) | 0.0 (1.0) at 112° | 0.4 (1.2) at 20° |
|  | *Anatomical* | 0.2 (1.4) | -0.4 (1.4) | 0.0 (1.1) at 82° | -0.2 (0.7) at 112° |
|  | *Oversize* | -0.2 (3.1) | -0.2 (3.1) | 0.0 (1.2) at 100° | -0.2 (2.9) at 43° |
|  | ***Anterior(+)/posterior(-) (mm)*** |  |  |  |  |
|  | *Undersize* | -1.0 (1.9) | -1.3 (1.9) | -1.0 (1.9) at 20° | -1.5 (0.8) at 80° |
|  | *Anatomical* | -0.8 (2.7) | -0.8 (2.7) | -0.7 (3.3) at 49° | -1.1 (3.3) at 83° |
|  | *Oversize* | -0.6 (5.2) | -0.9 (5.2) | -0.6 (5.2) at 20° | -1.5 (2.5) at 74° |
|  | ***Radial(+)/ulnar(-) (mm)*** |  |  |  |  |
|  | *Undersize* | -2.0 (1.8) | -1.5 (1.8) | -1.4 (1.0) at 103° | -2.1 (2.2) at 23° |
|  | *Anatomical* | -1.9 (1.2) | -1.3 (1.2) | -1.2 (0.9) at 101° | -2.0 (1.5) at 23° |
|  | *Oversize* | -1.1 (1.5) | -0.9 (1.5) | -0.8 (1.9) at 86° | -1.2 (1.6) at 43° |
| **Valgus loaded** | |  |  |  |  |
|  | ***Varus(+)/valgus(-) (deg)*** |  |  |  |  |
|  | *Undersize* | -0.0 (1.3) | -0.9 (1.3) | -0.0 (1.3) at 20° | -1.0 (1.3) at 108° |
|  | *Anatomical* | 0.2 (3.0) | -0.3 (3.0) | -0.0 (2.0) at 35° | -0.4 (0.9) at 80° |
|  | *Oversize* | -0.2 (2.5) | -0.5 (2.5) | -0.0 (2.1) at 21° | -0.9 (1.9) at 55° |
|  | ***Anterior(+)/posterior(-) (mm)*** |  |  |  |  |
|  | *Undersize* | -0.7 (2.0) | -1.0 (2.0) | -0.6 (1.6) at 39° | -1.0 (1.8) at 104° |
|  | *Anatomical* | -0.9 (3.2) | -0.6 (3.2) | -0.6 (3.0) at 112° | -0.9 (3.5) at 66° |
|  | *Oversize* | -1.0 (5.6) | -0.5 (5.6) | -0.5 (1.2) at 111° | -1.1 (3.8) at 48° |
|  | ***Radial(+)/ulnar(-) (mm)*** |  |  |  |  |
|  | *Undersize* | -1.1 (1.1) | -1.1 (1.1) | -0.8 (1.8) at 56° | -1.1 (1.1) at 20° |
|  | *Anatomical* | -1.4 (0.8) | -1.2 (0.8) | -0.9 (0.9) at 54° | -1.4 (1.1) at 28° |
|  | *Oversize* | -0.7 (0.7) | -0.7 (0.7) | -0.4 (0.8) at 57° | -0.9 (1.3) at 27° |

**Supporting table 3. Kinematic differences between native radial head and radius with RHA in supinated position.**

Mean and standard deviation of the differences between the native radial head and the radius with RHA with undersized, anatomical and oversized diameter for the kinematic parameters: varus/valgus angle, anterior/posterior shift and radial/ulnar shift. The 20° flexion angle represents kinematic difference at the start of the elbow flexion-extension motion, and the 120° flexion angle represents the end of the elbow flexion-extension motion. The smallest (min.) and largest (max.) difference between the native radial head and the radius with RHA are also presented. The color code corresponds to the color of the radius with RHA in figure 3, 4, 5 and 6.

|  | | **20° flexion angle (SD)** | **120° flexion angle (SD)** | **Min. (SD) at which flexion angle** | **Max. (SD) at which flexion angle** |
| --- | --- | --- | --- | --- | --- |
| **Unloaded** | |  |  |  |  |
|  | ***Varus(+)/valgus(-) (deg)*** |  |  |  |  |
|  | *Undersize* | 1.2 (1.6) | 1.5 (1.6) | 0.9 (1.6) at 60° | 1.7 (1.9) at 95° |
|  | *Anatomical* | 1.1 (1.2) | 1.0 (1.2) | 0.8 (2.1) at 61° | 1.1 (1.3) at 41° |
|  | *Oversize* | -0.0 (3.9) | 0.6 (3.9) | 0.0 (3.9) at 21° | 0.7 (2.2) at 108° |
|  | ***Anterior(+)/posterior(-) (mm)*** |  |  |  |  |
|  | *Undersize* | -0.6 (6.8) | -0.6 (6.8) | -0.6 (4.7) at 96° | -1.2 (9.4) at 53° |
|  | *Anatomical* | 0.3 (6.4) | 0.3 (6.4) | -0.0 (6.8) at 54° | 0.5 (3.2) at 85° |
|  | *Oversize* | 1.3 (6.0) | 0.4 (6.0) | 0.4 (5.2) at 112° | 1.3 (6.0) at 20° |
|  | ***Radial(+)/ulnar(-) (mm)*** |  |  |  |  |
|  | *Undersize* | -0.6 (2.2) | 0.3 (2.2) | -0.0 (1.2) at 70° | -0.7 (1.0) at 37° |
|  | *Anatomical* | 0.4 (2.3) | 1.2 (2.3) | 0.3 (2.3) at 25° | 1.2 (1.9) at 111° |
|  | *Oversize* | 1.3 (1.9) | 1.9 (1.9) | 1.1 (1.3) at 33° | 2.0 (2.1) at 108° |
| **Varus loaded** | |  |  |  |  |
|  | ***Varus(+)/valgus(-) (deg)*** |  |  |  |  |
|  | *Undersize* | 2.4 (2.2) | 1.9 (2.2) | 1.9 (2.3) at 112° | 2.4 (2.2) at 20° |
|  | *Anatomical* | 1.8 (3.1) | 1.5 (3.1) | 1.5 (2.4) at 48° | 1.9 (3.1) at 21° |
|  | *Oversize* | 0.3 (5.9) | 1.3 (5.9) | 0.2 (5.4) at 29° | 1.4 (5.0) at 110° |
|  | ***Anterior(+)/posterior(-) (mm)*** |  |  |  |  |
|  | *Undersize* | -0.3 (7.2) | -0.4 (7.2) | -0.3 (6.6) at 24° | -0.5 (5.1) at 24° |
|  | *Anatomical* | 0.4 (6.1) | 0.3 (6.1) | 0.3 (3.0) at 112° | 0.4 (1.8) at 31° |
|  | *Oversize* | 1.1 (5.6) | 0.5 (5.6) | 0.5 (3.8) at 99° | 1.1 (5.6) at 20° |
|  | ***Radial(+)/ulnar(-) (mm)*** |  |  |  |  |
|  | *Undersize* | -1.2 (1.7) | -0.2 (1.7) | -0.2 (1.1) at 112° | 0.0 (1.6) at 27° |
|  | *Anatomical* | 1.8 (3.1) | 1.5 (3.1) | 1.5 (2.4) at 48° | 1.9 (3.1) at 21° |
|  | *Oversize* | 1.4 (2.2) | 1.6 (2.2) | 1.2 (1.7) at 24° | 1.6 (1.2) at 69° |
